# The Regulation of DNA Damage Tolerance by Ubiquitin and Ubiquitin-Like Modifiers

**DOI:** 10.3389/fgene.2016.00105

**Published:** 2016-06-13

**Authors:** Lina Cipolla, Antonio Maffia, Federica Bertoletti, Simone Sabbioneda

**Affiliations:** Istituto di Genetica Molecolare, Consiglio Nazionale delle Ricerche, PaviaItalia

**Keywords:** DNA damage tolerance, translesion synthesis, ubiquitylation, SUMOylation, ISGylation, PCNA

## Abstract

DNA replication is an extremely complex process that needs to be executed in a highly accurate manner in order to propagate the genome. This task requires the coordination of a number of enzymatic activities and it is fragile and prone to arrest after DNA damage. DNA damage tolerance provides a last line of defense that allows completion of DNA replication in the presence of an unrepaired template. One of such mechanisms is called post-replication repair (PRR) and it is used by the cells to bypass highly distorted templates caused by damaged bases. PRR is extremely important for the cellular life and performs the bypass of the damage both in an error-free and in an error-prone manner. In light of these two possible outcomes, PRR needs to be tightly controlled in order to prevent the accumulation of mutations leading ultimately to genome instability. Post-translational modifications of PRR proteins provide the framework for this regulation with ubiquitylation and SUMOylation playing a pivotal role in choosing which pathway to activate, thus controlling the different outcomes of damage bypass. The proliferating cell nuclear antigen (PCNA), the DNA clamp for replicative polymerases, plays a central role in the regulation of damage tolerance and its modification by ubiquitin, and SUMO controls both the error-free and error-prone branches of PRR. Furthermore, a significant number of polymerases are involved in the bypass of DNA damage possess domains that can bind post-translational modifications and they are themselves target for ubiquitylation. In this review, we will focus on how ubiquitin and ubiquitin-like modifications can regulate the DNA damage tolerance systems and how they control the recruitment of different proteins to the replication fork.

## Introduction

DNA damage poses a constant threat to the genetic material. It can arise from products either of the cellular metabolism or by exposure to exogenous sources (physical or chemical). Regardless of its origin, DNA damage is addressed swiftly by the multitude of repair mechanisms that protect the integrity of the genome (Hoeijmakers, 2001). The DNA damage response provides an overall control network for the repair mechanisms and it allows the coordination of the complex biochemical reactions that lead to the elimination of DNA damage (Ciccia and Elledge, 2011). Unfortunately, in certain conditions, the cells are exposed to an amount of damage that the repair systems cannot handle completely. This could be caused either by an extreme insult, able to saturate one or multiple repair systems, or by damage that is repaired slowly. The result of both conditions is the permanence of lesions in the template DNA. Nevertheless, the damaged template then must be replicated during S phase. Replicative DNA polymerases are extremely efficient and processive but are unable to cope with a distorted template caused by DNA damage. To solve this impasse, cells possess damage tolerance pathways that are tasked with the bypass of the damage, which eventually will be repaired at a later stage (Sale et al., 2012). Failure to bypass the damage is believed to be one of the main causes of replication fork blocks, cell cycle arrest and eventually cell death.

During S phase, the damaged template can be replicated by either a special class of DNA polymerases, in a process called DNA translesion synthesis (TLS), or by a damage avoidance pathway that uses the sister chromatid as a template, in a mechanism called template switch. TLS utilizes specialized low-fidelity DNA polymerases (η, ι, κ, ζ, and Rev1), mostly belonging to the Y-family, to bypass the damaged template, while template switch is proposed to use a recombination-like mechanism. A crucial difference between the two pathways is that the former is potentially error-prone, while the latter is thought to be error-free (Branzei and Foiani, 2007; Sale et al., 2012). Given this background, the choice of pathway is extremely important in order to bypass the damage with the lowest possible chance of introducing mutations. Post-translational modifications play a central role in controlling damage tolerance and, in the last few years, emerging evidence has shown that ubiquitylation and SUMOylation sit at a crucial crossroad that influences its outcomes (Huang and D’Andrea, 2006; Bergink and Jentsch, 2009; Bekker-Jensen and Mailand, 2011; Mailand et al., 2013; Pinder et al., 2013).

Ubiquitylation is a process that involves the addition of ubiquitin to a target protein. This process is conserved in all eukaryotes and it controls a variety of cellular functions, ranging from protein degradation to cell cycle progression. Ubiquitylation is reversible and utilizes three classes of enzymes to target ubiquitin to a desired protein (Hershko and Ciechanover, 1998). In the initial step, an ubiquitin activating enzyme (E1) forms a thioester bond with ubiquitin. Afterward, ubiquitin conjugating enzymes (E2) transfer the ubiquitin from the E1 to the target protein, either directly or with the help of an E3 ubiquitin ligase that confers specificity to its E2 partner. Ubiquitin is normally attached via its C-terminus to lysines on the target proteins. Once ubiquitin has been linked to its target, it can be further modified by the addition of additional ubiquitin moieties on one of the lysines that can be found on ubiquitin itself: K6, K11, K27, K29, K33, K48, and K63 (Ikeda and Dikic, 2008; Kulathu and Komander, 2012). The linkage to the different lysines confers diverse structural properties to the polyubiquitin chains, creating a different binding platform for a variety of processes. For example, K48-linked chains have a compact structure (closed chain) and they direct proteins to degradation by the proteasome (Varadan et al., 2002). On the other hand, K63 chains are linear and flexible and they seem to have a more prominent role in mediating protein–protein interactions (Varadan et al., 2004). SUMOylation shares a similar activating pathway with ubiquitin but uses SUMO (Small Ubiquitin MOdifier) as a substrate (Muller et al., 2001; Hay, 2005). In most organisms, a single SUMO is present but human cells express 4 different variants (SUMO1–4, Hay, 2005). Remarkably, while in the human genome we can find between 10 to 35 ubiquitin E2s and hundreds of putativeE3 ubiquitin ligases have been predicted, this number is greatly reduced in the case of SUMO, up to the point where UBC9 encodes the only known SUMO E2 (Hay, 2005). The aim of this review is to highlight the crucial role of both ubiquitylation and SUMOylation in the regulation of the DNA damage tolerance pathways.

## Ubiquitylation of PCNA

A number of E2 and E3 enzymes has been known for a long time to be involved in the replication of damaged DNA, among these the proteins encoded by Rad6, Rad18, Ubc13, Mms2, and Rad5 in the yeast Saccharomyces cerevisiae (Jentsch et al., 1987; Bailly et al., 1994, 1997; Xiao et al., 2000). All of these proteins have been shown to ubiquitylate, in different ways, the PCNA, assigning to PCNA a central role in the regulation of damage bypass during replication (Hoege et al., 2002; Mailand et al., 2013).

**FIGURE 1:**
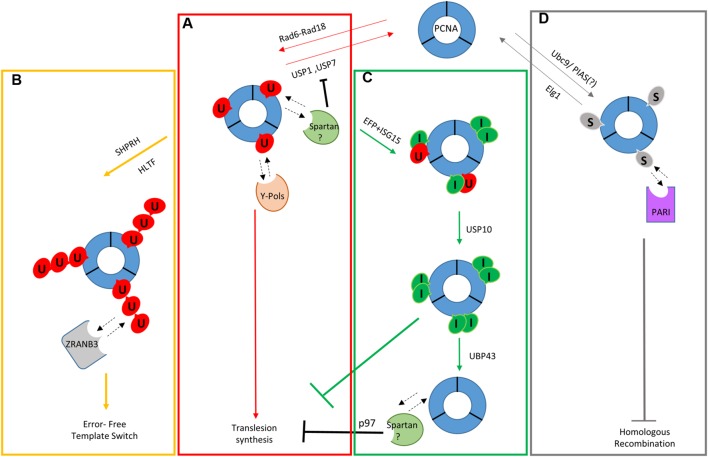
**Schematic model of ubiquitin and ubiquitin-like modifications in the DNA damage tolerance pathway. (A)** Monoubiquitylation of PCNA leading to TLS. **(B)** Polyubiquitylation of PCNA leading to template switch. **(C)** ISGylation of PCNA and recovery from TLS. **(D)** SUMOylation of PCNA during unperturbed S phase and inhibition of Homologous Recombination. Dotted lines indicate interactions between regulators of the DDT and modified/unmodified PCNA.

Proliferating cell nuclear antigen is a homotrimeric protein that acts as the processivity factor for DNA polymerases, in a role similar to *E. coli* β-clamp (Kuriyan and O’Donnell, 1993; Krishna et al., 1994a,b). Each subunit consists of two different domains connected by an interdomain connecting loop (IDCL). The IDCL makes contacts and tethers the DNA polymerases to the DNA. The binding to the IDCL of PCNA is mediated by a PCNA interacting peptide (PIP) motif present in the interacting partner. PCNA plays also crucial roles as a loading platform for a variety of proteins involved in different repair systems (Freudenthal et al., 2010; Dieckman et al., 2012). In yeast, PCNA was originally discovered to be ubiquitylated after the treatment with methyl methanesulfonate (MMS) by the complex formed by the ubiquitin ligase Rad18 and the ubiquitin conjugating enzyme Rad6 (Hoege et al., 2002) (**Figure 1**). Ubiquitylation was shown to be attached to lysine 164 that is located on the back side of the trimer, on the opposite side where the replicating polymerases make contact (front side, Freudenthal et al., 2010).

Once monoubiquitylated, PCNA (Ubi-PCNA) can be further modified resulting in the formation of K63-linked polyubiquitin chains (Hoege et al., 2002). The two modifications were proposed to channel the bypass toward different branches of damage tolerance, with monoubiquitylation leading to TLS and polyubiquitylation of PCNA steering the system toward template switch (Branzei, 2011; Giannattasio et al., 2014).

Orthologs of all the proteins involved in the process originally described in *S. cerevisiae* have been identified in both invertebrates and vertebrates and, overall, the system appears to be conserved across different organisms, although subtle differences are present. For example, in *Xenopus laevis*, PCNA is monoubiquitylated during an unperturbed S phase and this modification is required for the efficient progression of the replication fork in egg extracts, while polyubiquitylation of the trimer appears specifically only after DNA damage (Leach and Michael, 2005).

In vertebrates, the main modification of PCNA is monoubiquitylation. It is observed after treatments that block the progression of the replication fork (Kannouche and Lehmann, 2004; Kannouche et al., 2004; Watanabe et al., 2004). In such conditions, it is possible to detect an accumulation of single-stranded DNA (ssDNA), likely caused by the uncoupling of the activities of the blocked replication fork and the DNA helicase. At this point, RPA readily binds the free ssDNA creating the substrate for the recruitment of Rad18 and Rad6 that ubiquitylate PCNA on lysine 164 (Davies et al., 2008). Rad18 and replication protein A (RPA) interact directly and the recruitment of Rad6/Rad18 to RPA-coated ssDNA has been observed *in vitro* (Huttner and Ulrich, 2008). Monoubiquitylated PCNA has increased affinity for TLS polymerases, whose interactions are mediated by their PIP-boxes (PCNA-interacting peptide) and ubiquitin-binding motifs (Kannouche et al., 2004; Bienko et al., 2005; Dikic et al., 2009). Upon fork stalling, replicative polymerases slow down and dissociate from the replisome followed by the recruitment of TLS polymerases (polymerase switching; **Figure 1A**). In the last few years, there has been a progressive discovery of new factors that help Rad18 in promoting the efficient ubiquitylation of PCNA. One of these factors is a TLS polymerase itself. It is interesting to point out that originally the recruitment of TLS polymerases was proposed to be an event that followed the monoubiquitylation of PCNA. New experimental data seem to suggest that TLS polymerases can influence themselves the state of PCNA, and an increase in PCNA ubiquitylation has been observed, in some cell types, after polη overexpression (Durando et al., 2013; Masuda et al., 2015). In these conditions, polη is believed to enhance and stabilize Rad18 in the proximity of PCNA. Rad18 and polη have been purified as a stable complex and their interaction has been proposed to be dependent on the phosphorylation of Rad18. Rad18 is phosphorylated, at a basal level even in unperturbed conditions but this modification is enhanced after DNA damage by DDK (Dbf4/Drf1-dependent Cdc7 kinase) and JNK (c-Jun N-terminal kinase; Day et al., 2010; Barkley et al., 2012). This hyper-phosphorylation is believed to increase the affinity of Rad18 for polη and promote their mutual recruitment to the chromatin, leading to the ubiquitylation of PCNA. However, this model of action is still controversial since it would make the accumulation of Ubi-PCNA an event dependent on ATR and Chk1, in contrast with previous established experimental evidence that demonstrated that ubiquitylation of PCNA is independent from both ATM and ATR kinases and their respective DNA damage checkpoints (Chang et al., 2006; Davies et al., 2008; Gohler et al., 2008; Niimi et al., 2008; Yang et al., 2008). A cohort of new factors that have been found to interact with Rad18 to promote efficient PCNA ubiquitylation include NBS1 (Yanagihara et al., 2011), Claspin and Chk1 (Yang et al., 2008), RPA (Davies et al., 2008), Spartan (see later in this review, Centore et al., 2012; Davis et al., 2012; Juhasz et al., 2012; Mosbech et al., 2012) and SIVA1 (Han et al., 2014).

In human cells, Rad18 is the principal E3 ligase that monoubiquitylates PCNA, but avian DT40 cells lacking Rad18 (Rad18^-/-^) still show detectable levels of Ubi-PCNA, indicating the existence of another E3 ligase (Arakawa et al., 2006; Simpson et al., 2006). In fact, other minor pathways leading to the ubiquitylation of PCNA have been proposed also in *S. cerevisiae* and in human cells under specific conditions. In human cells, RNF8 and CRL4^Cdt2^ were identified as ubiquitin E3 ligases of PCNA, although their contribution is rather minor when compared to Rad18 (Zhang et al., 2008; Terai et al., 2010).

Rad18 is itself ubiquitylated and its modification is believed to control its availability and cellular localization. Rad18 has been reported to form a homodimer where the ubiquitin moiety on each Rad18 interacts with the UBZ (ubiquitin-binding zinc finger domain) of the other subunit (Miyase et al., 2005; Notenboom et al., 2007). Once Rad18 is de-ubiquitylated, it becomes active. The Rad18 dimer, which is considered inactive, is believed to localize mainly in the cytoplasm, while the active Rad18 monomer is distributed in the nucleoplasm. Recently, Rev1 has been shown to bind ubiquitylated Rad18 causing the release of non-modified Rad18 from the dimer, that is then free to ubiquitylate PCNA on the chromatin (Wang et al., 2016). This is another example of the extensive crosstalk between TLS polymerases, Rad18 and PCNA, further strengthening the idea that the regulation of DNA damage tolerance is far from a simple linear pathway.

Once ubiquitylated, PCNA can be further modified via K63-linked polyubiquitylation. In yeast, the complex formed by Ubc13-Mms2 (E2) and Rad5 (E3) is responsible for this modification (Hoege et al., 2002; Parker and Ulrich, 2009). In human cells, polyUbi-PCNA is hardly observed in comparison to yeast (Chiu et al., 2006) although all the proteins involved are believed to be conserved. Two Rad5 orthologs have been identified: helicase-like transcription factor (HLTF) and SNF2 histone linker PHD RING helicase (SHPRH; **Figure 1B**). HLTF is characterized by ATPase and HIRAN domains that promote fork regression *in vitro*, a crucial step in the stabilization of the replication fork in the presence of DNA damage (MacKay et al., 2009; Blastyak et al., 2010; Achar et al., 2015). Both HLTF and SHRPH can catalyze the addition of ubiquitin chains to Ubi-PCNA *in vitro* and their silencing, mediated by siRNA, results in a decrease in polyUbi-PCNA in living cells (Motegi et al., 2006, 2008; Unk et al., 2008, 2010). Recent evidence suggests that the loss of HLTF and SHPRH increases mutagenesis induced by UV and MMS treatment, respectively (Lin et al., 2011). HLTF has been shown to have also a role in the mono-ubiquitylation of PCNA and in the recruitment of polη (Lin et al., 2011). Surprisingly, mouse embyonic fibroblast (MEF) cells lacking both SHPRH and HLTF are still competent for PCNA polyubiquitylation and the double mutant is not hypersensitive to DNA-damaging agents (Krijger et al., 2011a). This seems to suggest the existence of yet another E3 ligase involved in PCNA ubiquitylation, at least in mouse. In light of all of this evidence, it is clear that further investigation will be required in order to understand the role of the Rad5 orthologs in higher eukaryotes.

## Going Back: The De-Ubiquitylating Enzymes

Ubi-PCNA plays a central role in the bypass of damaged DNA by facilitating the access of TLS polymerases to the replication fork. However, unscheduled recruitment of low-fidelity TLS polymerases would result in replication errors and mutagenesis on undamaged DNA, thus the level of Ubi-PCNA must be strictly controlled. Ubi-PCNA in human cells is negatively regulated by the ubiquitin-specific protease 1 (USP1; Huang et al., 2006) (**Figure 1A**). USP1 interacts with the activating protein partner UAF1 (USP1-associated factor 1) and de-ubiquitylate Ubi-PCNA in the absence of DNA damage (Cohn et al., 2007). USP1 is subjected to an auto-cleavage reaction, which regulates its cellular concentration (Cohn et al., 2007). Furthermore, high doses of UV-C light result in the down-regulation of the USP1 transcript, thus ensuring its down-regulation when the ubiquitylation of PCNA needs to be promoted (Huang et al., 2006). Indeed, Ubi-PCNA levels correlate nicely with the reduced expression levels of USP1 after UV treatment (Niimi et al., 2008). Differently from UV, USP1 is still present after hydroxyurea or MMS treatment, two genotoxic agents that induce a strong ubiquitylation of PCNA (Niimi et al., 2008). This observation suggests the possible presence of other negative regulators.

USP1 has been shown to protect the cells from genomic instability, as monitored by the formation of micronuclei, caused by the erroneous recruitment of polκ and the following decrease in fork progression (Jones et al., 2012). USP1 was the first and most prominent DUB involved in the negative regulation of PCNA ubiquitylation; however, recent data seem to suggest the involvement of more DUBs in the control of PCNA. Some of these DUBs either act directly on PCNA or can regulate other proteins that control its ubiquitylation. Among these, USP7, also called HAUSP, is the DUB that controls the stability of p53 by counteracting the activity of Mdm2, the E3 ligase responsible for its degradation (Li et al., 2002; Cummins and Vogelstein, 2004; Sheng et al., 2006). Recently USP7 has been shown to regulate indirectly the ubiquitylation of PCNA via the stabilization of either Rad18 or polη (Qian et al., 2015; Zlatanou et al., 2016). Other work has shown that USP7 can de-ubiquitylate Ubi-PCNA *in vitro* and it suppresses UV- and oxidative-stress-induced PCNA monoubiquitylation *in vivo* (Kashiwaba et al., 2015). PCNA ubiquitylation after DNA damage is normally very stable and can be detected days after the original genotoxic treatment (Niimi et al., 2008). Another DUB involved in the de-ubiquitylation of PCNA is USP10. USP10 can interact directly with PCNA via its PIP box and its silencing results in increased Ubi-PCNA 24 h after UV irradiation (Park et al., 2014). The activity of USP10 is remarkably deferred compared with USP1 as no difference could be appreciated in the levels Ubi-PCNA at 0 and 12 h after UV irradiation (Park et al., 2014), whereas silencing of USP1 results in the accumulation of Ubi-PCNA even in the absence of DNA damage (Huang et al., 2006). This seems to suggest that USP10 may control the de-ubiquitylation of Ubi-PCNA during the recovery from UV irradiation (see ISGylation, later on). An USP1 ortholog has not been identified in yeast. Recently, ubiquitin protease 10 (UBP10) was reported to de-ubiquitylate Ubi-PCNA in *S. cerevisiae* (Gallego-Sanchez et al., 2012). Cells lacking UBP10 accumulate Ubi-PCNA in response to DNA damage resulting in an increased interaction between PCNA and Rev1. UBP10 appears to de-ubiquitylate Ubi-PCNA during S phase and its protein levels remain constant after UV treatment suggesting that UBP10 in yeast and USP1 in human regulate the de-ubiquitylation of PCNA by different mechanisms (Gallego-Sanchez et al., 2012).

## New Readers of Ubiquitylated PCNA

Once PCNA is ubiquitylated, it provides a loading platform for a variety of proteins involved in the replication of damaged DNA. As already mentioned, Ubi-PCNA can recruit a plethora of TLS polymerases allowing damage bypass and the restart of a stalled replication fork (Sale et al., 2012). Recently at least two new proteins have been described to be able to read the state of ubiquitylated PCNA and to help in maintaining the stability of the fork: Spartan, also called DVC1, and ZRANB3 (Centore et al., 2012; Davis et al., 2012; Mosbech et al., 2012) (**Figures 1A–C**).

Spartan is a substrate of the anaphase promoting complex and localizes to replication factories in a manner dependent on both its PIP and UBZ domains (Davis et al., 2012; Mosbech et al., 2012). In its absence, cells become hypersensitive to DNA damage agents and they are deficient in the DNA damage tolerance (DDT) response. Spartan can bind to p97 via its SHP domain (Davis et al., 2012; Mosbech et al., 2012). p97 encodes for a chaperone protein that can remodel ubiquitylated proteins in an ATP-dependent manner (Meyer et al., 2012).

As mentioned, Spartan PIP box and UBZ domain are needed for its accrual in replication factories and DNA damage foci. While all the data in the literature consistently report that PCNA is required for Spartan recruitment, the role of Ubi-PCNA as the target of Spartan’s UBZ is still controversial. Spartan can bind Ubi-PCNA *in vitro* (Centore et al., 2012) but there are discording evidences that this may occur *in vivo*. Two groups reported that Spartan could relocalize to replication factories when Rad18 is depleted by siRNA, a condition that results in the absence of Ubi-PCNA (Davis et al., 2012; Mosbech et al., 2012). Spartan itself is ubiquitylated and this modification prevents further binding to ubiquitin targets and decreases its accumulation in focal structures (Centore et al., 2012).

Given all the conflicting evidence, the role of Spartan is still under scrutiny, with at least two proposed models of actions. In the first Spartan is thought to bind to Ubi-PCNA and to promote both Rad18 and polη recruitment to the chromatin. Its binding would shield Ubi-PCNA from being de-ubiquitylated by USP1 or by another DUB, and in its absence PCNA ubiquitylation appears to be reduced (Centore et al., 2012) (**Figure 1A**). At the opposite side of the spectrum, an alternative mechanism proposes Spartan acting as a negative regulator of TLS. In this scenario, Spartan is thought to recruit p97, which in turn will remove polη from the replication fork in order to resume processive replication (**Figure 1C**). This model is substantiated by increased focal retention of polη and increased mutagenesis when Spartan is silenced (Davis et al., 2012; Mosbech et al., 2012). Recently, three patients showing early onset hepatocellular carcinomas and progeroid syndrome have been found to carry a mutation in SPRTN (Lessel et al., 2014). When Spartan was mutated or depleted, the cells showed signs of genomic instability, defects in replication fork progression and cell proliferation. Interestingly, depletion of polη in a background mutated in SPRTN did not rescue the replication phenotypes, indicating that polη is potentially not the main target of Spartan activity (Lessel et al., 2014). The discovery of this new progeroid syndrome further stresses the importance of SPRTN, but additional investigation is needed to clarify the mechanism of action of this protein essential for the DDT.

Proliferating cell nuclear antigen polyubiquitylation is proposed to channel the DDT to an error-free damage avoidance branch named template switch (Hoege et al., 2002; Branzei and Foiani, 2007; Branzei, 2011). The molecular mechanism of this pathway is still not completely understood and, until recently, we did not know the role of K63-linked chains attached to PCNA. In the last couple of years the protein ZRANB3/AH2, has been proposed to be able to recognize specifically polyubiquitylated PCNA and to promote template switch by stimulating fork regression (Ciccia et al., 2012; Weston et al., 2012; Yuan et al., 2012). ZRANB3 encodes for an annealing helicase/translocase and it can interact with polyUbi-PCNA via multiple domains. A canonical PIP motif and an APIM (C-terminal AlkB2 PCNA-interaction motif) domain mediate the direct interaction with the PCNA trimer while an NPL4 zinc finger (NZF), a variant of ubiquitin-binding domain, recognizes K63-linked ubiquitin chains specifically (Ciccia et al., 2012). This domain is able to bind to polyUbi-PCNA *in vitro* and it is needed for the localization of ZRANB3 to damage sites. All these structural motifs are required for restarting the fork after DNA damage (**Figure 1B**).

Experimental observations suggest that ZRANB3 may play three different roles at the stalled replication fork: (1) it can stimulate fork regression in order to stabilize the fork and minimize the amount of ssDNA that is generated (Ciccia et al., 2012). (2) ZRANB3 can disrupt D-loop formation *in vitro* and this in turn could result in the prevention of inappropriate homologous recombination (HR) (Ciccia et al., 2012); (3) it can act as a strand-specific endonuclease pointing to a role not only in damage bypass but also in damage repair (Weston et al., 2012).

ZRANB3 may act in parallel or in conjunction with HLFT that also has a helicase activity and can stimulate fork regression *in vitro* (Blastyak et al., 2010; Achar et al., 2015). Further work will be needed in the future to completely elucidate ZRANB3 role in damage tolerance and repair.

## PCNA SUMOylation and ISGylation

Another prominent post-translational modification of PCNA is its SUMOylation. It was originally identified in yeast and only recently it was observed in human cells.

In yeast, PCNA is SUMOylated (S-PCNA) on Lys164 (major) and Lys127 (minor) by the combined action of Ubc9 (E2) and Siz2 (E3) or by Ubc9 alone, respectively (Hoege et al., 2002) (**Figure 1D**). SUMOylation occurs during normal S phase and/or in response to high doses of DNA damage (Juhasz et al., 2012). SUMOylated PCNA interacts with Srs2 helicase, which has been shown to prevent HR by disrupting Rad51 filaments (Papouli et al., 2005; Pfander et al., 2005). Srs2 has a non-canonical PIP-box with limited affinity for PCNA and it binds stably only when the clamp is SUMOylated. A SUMO interacting motif that is located in tandem after the PIP in the protein carboxyl terminus of Srs2 mediates this interaction (Kim S.O. et al., 2012).

Given the catalytic activity of Srs2 and the timing of this modification, it is believed that SUMOylation of PCNA acts as a negative regulator of unscheduled HR during S phase, where this kind of pathway could be detrimental to the cell. In yeast, one of the replication factor C (RFC)-like complexes, Elg1-RFC also has a role in regulating S-PCNA. RFC is a complex consisting of Rfc1-5 and it works as clamp loader/unloader. All eukaryotic cells contain a series of three alternative RFCs, containing Elg1, Ctf18, or Rad24 in place of Rfc1(Kim and MacNeill, 2003). Elg1-RFC is required for the efficient unloading of SUMOylated PCNA from the chromatin during S phase. In cells lacking Elg1, PCNA accumulates on the chromatin and it is possible to detect an increase in SUMOylated PCNA (Parnas et al., 2010; Kubota et al., 2013b).

In *X. laevis* S-PCNA is present during unperturbed replication in cell extracts, but it is not required for the replication of either ssDNA or sperm chromatin (Leach and Michael, 2005).

In human cells, S-PCNA had eluded detection for a number of years and it has been detected only recently after overexpression of SUMO1, although to a much less extent than the levels detected in yeast (Moldovan et al., 2012). PCNA was found to be SUMOylated on both Lys164 and Lys254 under specific conditions (Gali et al., 2012). As in yeast, mammalian UBC9 acts as the E2 enzyme but surprisingly, at least *in vitro*, the SUMOylation of PCNA does not require the Siz1 orthologs (PIAS1-4) in either lysine residues (Gali et al., 2012).

A PCNA-SUMO fusion protein not only prevents HR, but also DNA double-strand break formation, as monitored by a marked reduction of γH2AX foci (Gali et al., 2012). Two putative functional homologs of Srs2 have been identified in human cells: PCNA-associated recombination inhibitor (PARI; Moldovan et al., 2012) and F-box DNA helicase (FBH1; Fugger et al., 2009; Bacquin et al., 2013). Both PARI and FBH1 have been reported to interact with PCNA and to have PCNA-dependent anti-recombinogenic activity, but only PARI seems to specifically interact with SUMOylated PCNA, at least *in vitro* (Moldovan et al., 2012). On the other hand, FBH1 needs to be degraded, via CRL4^Cdt2^ pathway in order to allow efficient recruitment of polη to replication factories (Bacquin et al., 2013).

In human cells, ATAD5, the ortholog of yeast Elg1 appears to have a somehow different role from its yeast counterpart as it interacts, at stalled replication forks, with the USP1/UAF1 complex and facilitates USP1-mediated PCNA de-ubiquitylation (Lee et al., 2010; Kubota et al., 2013a).

Last year ISGyaltion, another ubiquitin-like modification, was discovered to affect PCNA.

ISG15 (interferon-stimulated gene 15) was the first identified ubiquitin-like protein and it is strongly stimulated by type I interferon (Haas et al., 1987; Loeb and Haas, 1992). As ubiquitin and SUMO this post-translational modification relies on a chain of three classes of enzymes to be linked to its substrates: UBE1L is the activating E1 enzyme, followed by UBCH8 (E2) and finally by EFP and HERC5 (E3s; Yuan and Krug, 2001; Kim et al., 2004; Zhao et al., 2004; Dastur et al., 2006; Zou and Zhang, 2006). PCNA was reported to be bi-ISGylated 24 h after UV irradiation by EFP on both K164 and K168 (Park et al., 2014). Mutations of either residues resulted in the complete disappearance of ISGylated PCNA indicating that ISGylation at one site influences the state of the other. The late response to UV irradiation suggested that ISG15 had a role in the recovery from DNA damage and post-replication repair (PRR). The E3 ligase EFP interacts with Ubi-PCNA and this interaction is propaedeutic to PCNA ISGylation (Park et al., 2014). This modification in turn recruits USP10 that de-ubiquitylates PCNA in order to block TLS and resume normal replication. Eventually, UBP43 removes ISG15 from PCNA (**Figure 1C**). ISGylation-deficient mutants of PCNA show increased recruitment of polη to the chromatin many hours after UV irradiation (Park et al., 2014).

## Ubiquitylation of TLS Polymerases

As mentioned before, PCNA is not the only player that is modified in order to control PRR. All the members of the Y-family of DNA polymerase (η, ι, κ, and Rev1) involved in DNA TLS have been identified to be modified by ubiquitin or ubiquitin-like modifiers (Sale et al., 2012). Furthermore, all four of them contain ubiquitin-binding domains (UBM or UBZ; (Bienko et al., 2005; Guo et al., 2006, 2008; Plosky et al., 2006).

Probably, the best characterized of the group is polη, the major TLS polymerase involved in the error-free bypass of cyclobutane pyrimidine dimers (CPDs), the main adduct created by UV irradiation. CPDs are repaired slowly by the nucleotide excision repair (NER) and have a higher probability to persist in the genome until DNA replication. The importance of the bypass performed by polη is exemplified by the fact that individuals carrying an inactivating mutation are affected by *Xeroderma pigmentosum* Variant (XPV; Masutani et al., 1999). Regardless of the importance of its function, polη shares a common characteristic with other Y-family polymerases, a wide catalytic site. This structural feature, while beneficial for damage bypass, makes the polymerase intrinsically error-prone compared to replicating polymerases when using undamaged DNA as a template. For this reason, its recruitment to the replication fork needs to be tightly regulated. Polη is recruited to replication factories in a manner dependent on its PIP-box and UBZ, a specialized ubiquitin-binding zinc finger (Kannouche et al., 2001, 2002; Bienko et al., 2005, 2010; Sabbioneda et al., 2009). The presence of both domains stabilizes the interaction between the polymerase and Ubi-PCNA after DNA damage (Kannouche et al., 2004; Bienko et al., 2010). Mutants in either the PIP-box or the UBZ are required for focal accumulation of the polymerase but they retain a partial bypass activity, indicating that they work in parallel to ensure efficient binding with PCNA (Bienko et al., 2010). Ubiquitylation of PCNA provides a positive regulation by increasing the affinity between polη and the clamp when the replication fork is blocked (Kannouche et al., 2004).

Conversely, ubiquitylation of the polymerase works as a negative regulator by preventing its recruitment on the chromatin (Bienko et al., 2010). *In vivo*, a small amount of polη is monoubiquitylated, in the absence of damage, in its nuclear localization signal directly adjacent the PIP-box. The modification occurs primarily on K682 but in its absence, also K686, K694 and K709 have been found to be ubiquitylated (Bienko et al., 2010; Jung et al., 2011). Ubiquitylation is strictly dependent on the UBZ of polη. Recently, PirH2 was discovered to be the E3 ligase responsible for this monoubiquitylation (Jung et al., 2011). Ubiquitylation of polη is believed to cause a conformational change in its C-terminus with the attached ubiquitin binding intra-molecularly to polη’s UBZ. In this closed confirmation, neither the UBZ, blocked by the binding to the ubiquitin attached to polη, nor the PIP-box, that is located between the UBZ and K682, are available to stabilize its interaction with PCNA (Bienko et al., 2010). Ubi-polη is indeed excluded from the chromatin and replication foci. After DNA damage, ubiquitylated polη gradually disappears. The polymerase can be then recruited to the chromatin and it becomes proficient for TLS. The de-ubiquitylation of the polymerase is believed to be carried out by the DUB USP7 (Qian et al., 2015). It is important to note that only 10% of polη is ubiquitylated in the absence of damage at any given time, indicating that some other forms of regulation are keeping polη under negative control. In some cellular background, polη gradually disappears in the hours following UV irradiation. This process is believed to be mediated by Mdm2 that polyubiquitylate the polymerase and marks it for proteasomal degradation (Jung et al., 2012). A similar system, mediated by CRL4^Cdt2^ has also been observed in *Caenorhabditis elegans*. Interestingly in this system, the degradation of polη is prevented by its SUMOylation by the SUMO E3 ligase GEI-17 (Kim and Michael, 2008). It is still unclear whether polη is SUMOylated in human cells.

Similarly, to polη also its paralog polι is ubiquitylated (Bienko et al., 2005; McIntyre et al., 2015). This polymerase is thought to bypass lesions when polη is not present (Wang et al., 2007; Vidal and Woodgate, 2009). *In vitro*, polι can bypass different typologies of DNA adducts with different degrees of fidelity (Washington et al., 2004a,b; Frank and Woodgate, 2007).

Polι is characterized by two UBMs that are needed for its modification and correct localization in replication foci (Bienko et al., 2005; Bomar et al., 2010). It is speculated that the ubiquitylation of polι might be important for its interaction with polη (McIntyre et al., 2013).

The deoxycytidyl transferase Rev1 possesses two UBMs (Bomar et al., 2010) and gets ubiquitylated *in vivo* (Guo et al., 2006; Kim H. et al., 2012). The UBMs are needed for the efficient interaction with Ubi-PCNA (Guo et al., 2006; Wood et al., 2007). In yeast, deletion of UBM2 severely affects UV-induced mutagenesis, a pathway that is strictly dependent on TLS (Wood et al., 2007; Terai et al., 2010). Mutations in Rev1’s UBMs make the cells hypersensitive to UV in the DT40 system (Guo et al., 2006). In chicken cells, Rev1 and its UBMs have been shown to have a role in replication fork progression in the presence of UV in a process that is independent from Ubi-PCNA (Edmunds et al., 2008). Finally, Rev1 appears to be able to bind to the Fanconi core complex via FAAP20 and this interaction is believed to promote Rev1 recruitment to replication foci and ultimately Rev1-dependent mutagenesis (Mirchandani et al., 2008; Kim H. et al., 2012).

The last TLS polymerase that has been reported to be ubiquitylated is polκ (Guo et al., 2008). Polκ is characterized by two UBZ domains in its c-terminus (Bienko et al., 2005) that have been reported to be important for the interaction with PCNA and the localization in foci after UV irradiation (Guo et al., 2008). Polκ has also been shown to be important for NER, and its repair function depends on its UBZs (Ogi et al., 2010).

The role of polκ ubiquitylation is currently not clear but it is likely to promote protein–protein interaction similarly to the other members of the Y-family of DNA polymerases.

## DNA Damage Tolerance and Cancer

Post-replication repair and the damage tolerance systems provide an essential safety mechanism that allows the completion of DNA replication and it is an important pathway to preserve genome stability. At the same time, it can act as a double-edged sword since a number of its components, such as TLS polymerases, are intrinsically error prone and can be a source of mutations if they are not correctly regulated. Mutations are one of the major driving forces that lead to cell transformation and tumorigenesis, therefore it is important to define the contribution of PRR in the context of cancer. The dichotomy of protection versus increased risk is emblematic in the case of polη. As already mentioned in this review, a deficiency in polη is the cause of XPV (Broughton et al., 2002). Like other XP groups that are mutated in NER, XPV patients are sensitive to sun light and are extremely prone to both melanoma and non-melanoma skin cancers (Fassihi et al., 2016). Polη is the main polymerase that is able to bypass CPDs in an error-free manner and it possible to envisage that when missing, its role is carried out by other TLS polymerases with different degrees of fidelity.

In these cases, the ultimate and less than desirable outcome would be the introduction of mutations that are responsible for the transformation of the skin cells. It is important to note that polη-deficient patients are the most prone to skin cancers among the *X. pigmentosum* groups (Fassihi et al., 2016). XPV patients tend to have milder skin phenotypes and are normally diagnosed much later in their life, when they have already accumulated a number of UV-induced mutations. This higher mutation load correlates with the possibility of developing more skin tumors in their adult life (Fassihi et al., 2016). In this context, it is clear that polη protects the cells from cancer. On the other hand, the survival capability conferred by this polymerase can be hijacked to make tumors more resilient. *In vitro*, cells lacking polη are more sensitive to cisplatin, one of the most used first line drug in chemotherapy (Albertella et al., 2005a). Increased expression of polη associates with worse prognosis and survival in a cohort of patients suffering from non-small cell lung cancer patients previously treated with platinum (Ceppi et al., 2009). Polη seems also to be involved in the cellular response after treatment with nucleoside analogs, which are commonly used in the clinic as cancer drugs (Chen et al., 2006). Interestingly, mutations in polη are hardly found in patients with sporadic skin carcinomas (Glick et al., 2006; Flanagan et al., 2007; Lange et al., 2011) but its overexpression has been reported (Albertella et al., 2005b). Polη ortholog, polι, has been found to be elevated in breast cancer cells and in these cell lines a reduced mutation frequency was recorded when the polymerase was depleted *in vitro* (Yang et al., 2004). Furthermore, mutation in polι have been linked to an increased predisposition of developing lung cancer in both human (Sakiyama et al., 2005) and mouse (Wang et al., 2004; Lee and Matsushita, 2005).

Two of TLS polymerases extensively characterized for their role in mutagenesis and cancer are polζ and Rev1. Polζ is thought to be the major player involved in error-prone replication of damaged templates *in vivo*. In mice, conditional Rev3 knockout results in increased genome instability and tumorigenesis in a p53-null background (Wittschieben et al., 2006, 2010; Lange et al., 2013). Similarly to polη, there is experimental evidence indicating that the presence of both Rev1 and polζ can confer drug resistance both *in vitro* and *in vivo* (Xie et al., 2010). Conversely, Rev3 inhibition makes lymphoma and lung cancer cells more sensitive to platinum-derived drugs (Doles et al., 2010), once again underlying the dichotomy of TLS regarding cancer and genome protection. All of these evidences point to the idea that transient inhibition of TLS could be synthetically lethal to tumor cells that rely on the TLS mutator activity for survival. TLS polymerases are not the only proteins involved in damage tolerance that have been linked to cancer development. The expression of the E3 ligase HLTF has been found to be altered in transformed cells and in numerous tumors. A reduced expression of HLTF, due to hyper-methylation of its promoter, has been found in colon and colorectal cancer, esophageal squamous cell and gastric carcinomas (Debauve et al., 2008). Interestingly HLTF is overexpressed in transformed cells, indicating that a differential modulation of its expression could be needed at different stages of tumorigenesis (Debauve et al., 2008). Given the role of HLTF in the control of the error-free branch of damage tolerance, it is tempting to speculate that it could be beneficial for tumor cells to inactivate HLTF in order to channel the PRR pathway toward the more mutagenic TLS bypass, thus allowing the malignant cells to accumulate more mutations. As mentioned before a SPRTN deficiency has been linked with a new progeroid syndrome with propensity to develop early onset hepatocellular carcinomas, but it is still not clear whether this phenotype is directly linked with its proposed control of polη (Lessel et al., 2014). In conclusion, a tight regulation of TLS and the DNA damage tolerance pathway in general is required to preserve the delicate balance between protecting the genome stability and inducing cellular transformation.

## The Unanswered Questions

In the last decade, mounting evidence has pointed out the crucial role of ubiquitin, and other ubiquitin-like modifications, in the control of PCNA and TLS. Nevertheless, we still do not know whether PCNA ubiquitylation is strictly required for TLS. A series of experimental hints suggest that there is more to the story and we still have only a partial picture of the regulation of the damage tolerance pathway. For instance, MEF cells carrying the PCNA K164R mutation can be further sensitized by the deletion of other TLS genes, indicating that some steps of the pathway could be independent from Ubi-PCNA (Hendel et al., 2011). Furthermore, PCNA ubiquitylation is not required for polη-mediated somatic hyper-mutation in mouse B cells (Krijger et al., 2011b).

In human cells the phosphorylation of polη, that occurs on the chromatin, is dependent on its UBZ, indicating that the binding to ubiquitin is needed for this regulatory modification (Gohler et al., 2011). However, this phosphorylation does not require Ubi-PCNA and can occur in its absence (Gohler et al., 2011). Dynamic studies on polη show that Ubi-PCNA helps in stabilizing the polymerase in replication foci but do not exclude the possibility that other ubiquitylated proteins may play a role in its initial recruitment (Sabbioneda et al., 2008). Consistent with this hypothesis polη is still recruited to replication factories after chemical depletion of Ubi-PCNA caused by prolonged treatment with the proteasome inhibitors MG132 or epoxomicin (Sabbioneda et al., 2008). It must be noted that mouse cells carrying a homozygous K164R mutation appear to be deficient for polη recruitment (Krijger et al., 2011b), and so far no explanation has been found for these conflicting evidences.

## Conclusion

We are now starting to grasp the complexities of the regulation of PRR and TLS, the continuous dance between protein partners and the intricacies that lie behind such an important tolerance pathway. Meanwhile, behind the scenes, the hunt for the next big ubiquitylated/SUMOylated target still rages on.

## Author Contributions

All authors listed, have made substantial, direct and intellectual contribution to the work, and approved it for publication.

## Conflict of Interest Statement

The authors declare that the research was conducted in the absence of any commercial or financial relationships that could be construed as a potential conflict of interest.
