# Erratum: The Regulation of DNA Damage Tolerance by Ubiquitin and Ubiquitin-Like Modifiers

**DOI:** 10.3389/fgene.2016.00184

**Published:** 2016-09-30

**Authors:** Frontiers Production Office

**Keywords:** DNA damage tolerance, translesion synthesis, ubiquitylation, SUMOylation, ISGylation, PCNA

Reason for Erratum:

Due to the typesetting error, the Greek symbols (**ζ**, **η**, **κ**, **ι**) got mixed-up throughout the proof in the original publication. The corrections have been made in all the corresponding instances as mentioned in the table below, also it has been highlighted in the supplementary material for reader's convenience.

The publisher apologizes for this error, and this error does not change the scientific conclusions of the article in any way.

The original article has been updated.

**Table:** 

**Line number**	**Changed from**	**Changed to**
421	Polη	polκ
719	Polη	polι
722	Polη	polι
725	Polη	polι
728	Polη	polι
747	Polη (both the occurrences)	polκ
751	Polη	polκ
753	Polη	polκ
804	Polη	polι
807	Polη	polι
812	Polη (both the occurrences)	Polζ
818	Polη	Polζ

